# Supplementary material for: Therapeutic targeting of the PLK1-PRC1-axis triggers cell death in genomically silent childhood cancer
Source: Nat Commun. 2021 Sep 16;12:5356. doi: 10.1038/s41467-021-25553-z (PMC8445938; doi:10.1038/s41467-021-25553-z)
Supplement: Supplementary file 1 — Supplementary Information [file 41467_2021_25553_MOESM1_ESM.pdf]

Supplementary Figure 1 Li *et al.*

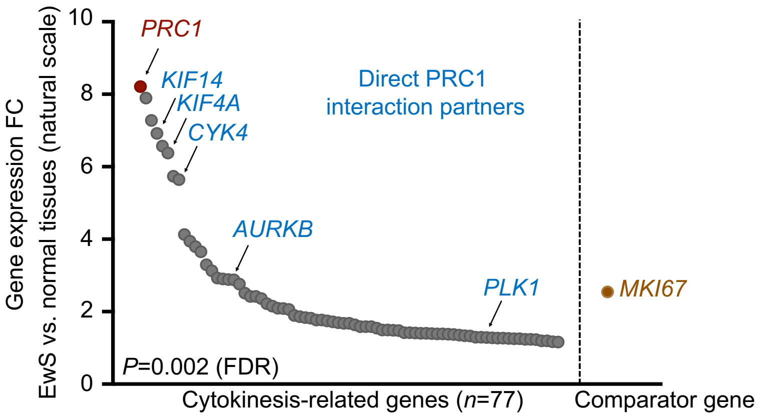

Supplementary Fig. 1 | *PRC1* is the top overexpressed cytokinesis-related gene in EwS
Displayed are 77 significantly upregulated genes in EwS compared to normal tissues (linear fold change (FC)>1, FDR P<0.05) that are annotated for the GO-term cytokinesis (GO:0000910). *MKI67* encoding the proliferation marker Ki-67 was used as a comparator. *PRC1* and its direct interacting/binding partners, such as cytokinesis defect 4 (*CYK4*), aurora kinase B (*AURKB*), the kinesin family members 14 and 4A (*KIF14/4A*) and *PLK1*, have been indicated by arrows. FDR (false discover rate) multiple test.

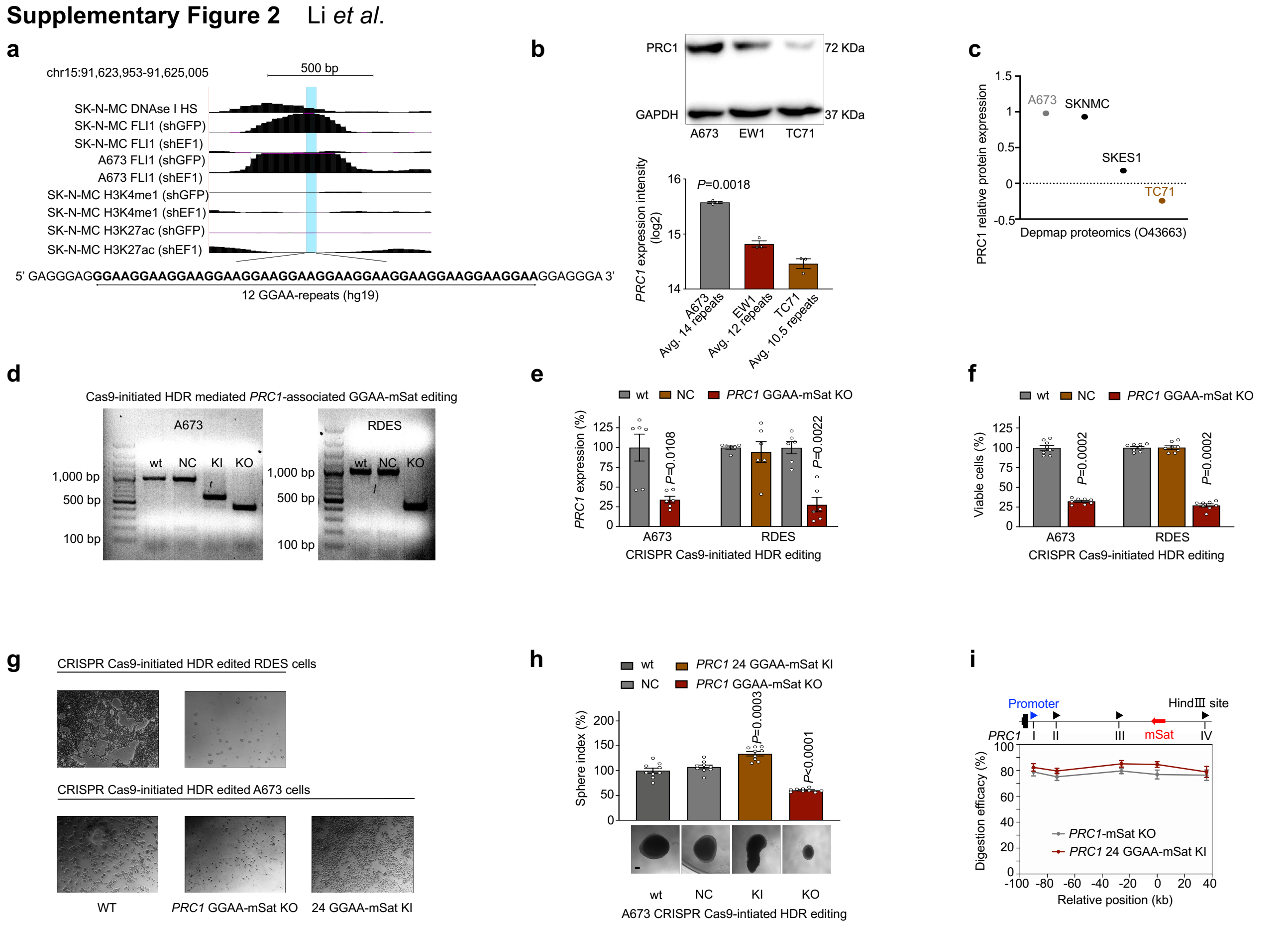

Supplementary Fig. 2 | Physical interaction of the *PRC1*-associated GGAA-mSat with EWSR1-FLI1 regulates *PRC1* expression and induces morphological changes

a) Integrative genomic view (hg19) of the cloned fragment within the *PRC1* locus from EwS cells expressing shRNAs targeting either *GFP* (shGFP; control) or *EWSR1-FLI1* (shEF1); blue = *PRC1*-associated GGAA-mSat. b) Upper: Representative western blot of indicated EwS cell lines. GAPDH = loading control. Lower: Corresponding *PRC1* expression intensity (Affymetrix Clariom D microarrays, triplicates per cell line). The average number of GGAA-repeats at the *PRC1*-associated GGAA-mSat is reported. Horizontal bars represent means and whiskers SEM. One-way ANOVA test. c) Analysis of relative PRC1 expression from the DepMap proteomics (O43663) dataset comprising 4 EwS cell lines. EwS cell lines A673 and TC71 corresponding to c) are indicated in gray or brown color, respectively. Data censoring: 25th May 2021. d) Representative images (from 3 independent experiments) of agarose gelelectrophoresis of amplicons containing the wildtype (wt) *PRC1*-associated GGAA-mSat, CRISPR Cas9-edited negative control (NC), or CRISPR Cas9-initiated HDR edited KO of A673 and RDES cells, and the insertion of 24-GGAA-repeats in A673 cells. e) Analysis of *PRC1* expression by qRT-PCR in A673 and RDES wt cells, CRISPR Cas9-initiated HDR edited NC, and *PRC1*-associated GGAA-mSat KO cells. Data are displayed as individual dots. Horizontal bars represent means and whiskers SEM, n=6 biologically independent experiments; Two-sided Mann-Whitney test. f) Proliferation analysis of A673 and RDES wt, CRISPR Cas9-initiated HDR edited NC and *PRC1*-associated GGAA-mSat KO cells 72h after seeding. Data are displayed as individual dots. Horizontal bars represent means and whiskers SEM, n=8 biologically independent experiments; two-sided Mann-Whitney test. g) Representative cell culture images of A673 and RDES wt cells and their CIRPSR Cas9-initiated HDR derivatives (magnification: 40×). h) Sphere formation of A673 wt and their CRISPR/Cas9-initiated HDR edited *PRC1*-associated GGAA-mSat derivatives. Data are displayed as individual dots. Horizontal bars represent means and whiskers SEM, n=9

biologically independent experiments; two-sided Mann-Whitney test. Representative images of spheres are shown; scale bar=50 µm. i) Digestion efficiency for the HindIII digested fragments analyzed in the 3C-PCR assays measured by qRT-PCR. Dots represent means and whiskers SEM, *n*=4 biologically independent experiments.

Supplementary Figure 3 Li *et al.*

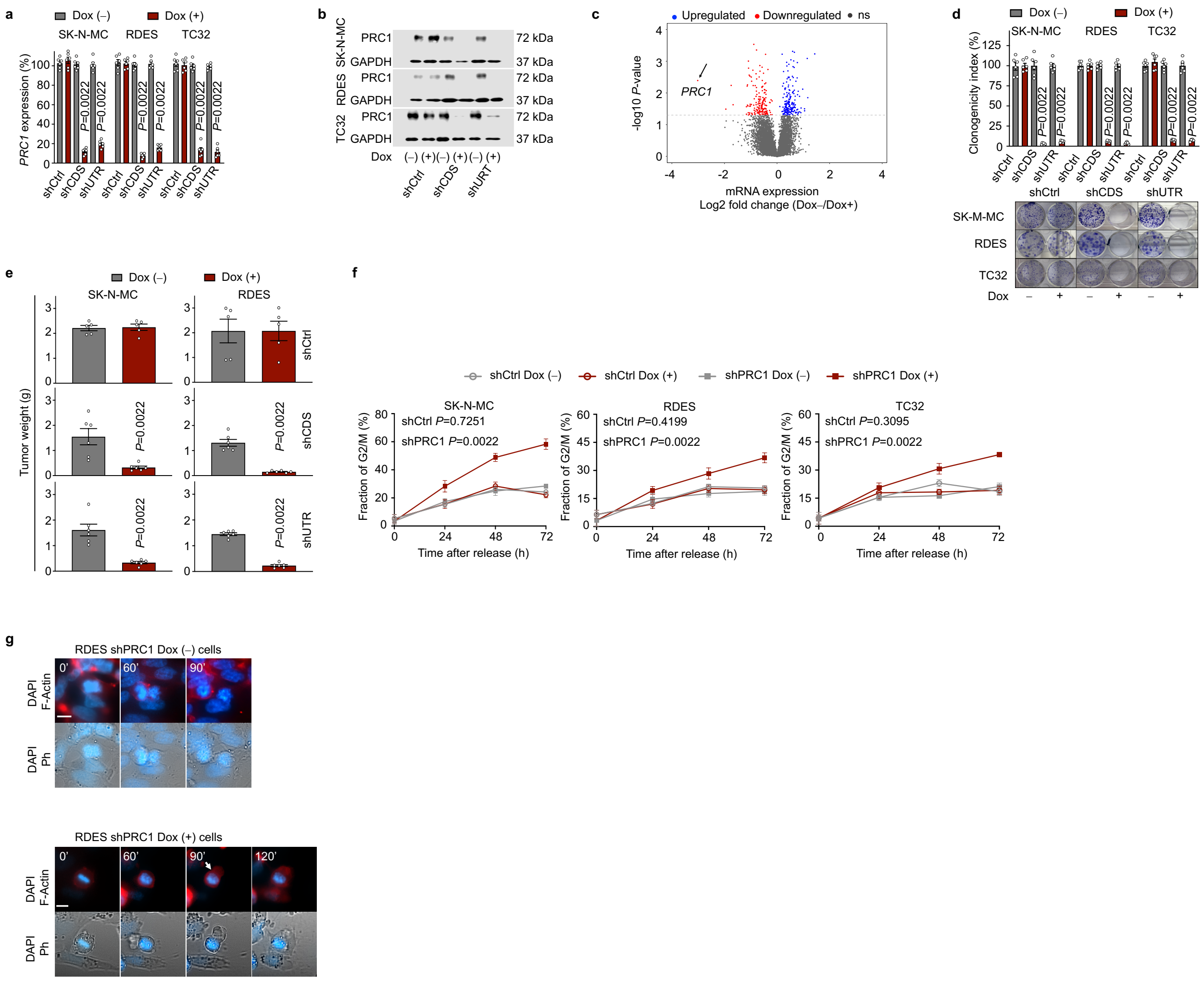

Supplementary Fig. 3 | PRC1 promotes EwS proliferation and growth

a) Analysis of *PRC1* mRNA levels by qRT-PCR in three EwS cell lines harboring Dox-inducible shPRC1 (shCDS/shUTR) or shCtrl constructs. Data are displayed as individual dots. Horizontal bars represent means and whiskers SEM, *n*=6 biologically independent experiments. b) Representative images of Western blots of cells depicted in (a). GAPDH served as loading control. c) Volcano-plot depicting differentially expressed genes of RDES and SK-N-MC cells with/without *PRC1* knockdown (summary level data). Arrow indicates *PRC1*. d) Colony-forming assays of EwS cells with Dox-inducible *PRC1* knockdown or a non-targeting shCtrl. Data are displayed as individual dots. Horizontal bars represent means and whiskers SEM, *n*=6 biologically independent experiments. e) Tumor weight (g) of xenografts from SK-N-MC and RDES cells containing two Dox-inducible shPRC1 constructs (shCDS/shUTR) or a non-targeting shCtrl. Data are displayed as individual dots. Horizontal bars represent means and whiskers SEM, *n* indicates the number of animals per condition: *n*=5 for shCtrl in both cell lines, *n*=6 for shCDS and shUTR in both cell lines. f) Analysis of G2/M cell cycle distribution after knockdown of *PRC1* in synchronized EwS cells harboring Dox-inducible shPRC1 or shCtrl constructs at 0h, 24h, 48h and 72h after releasing from G1/S blockage. Dots represent means and whiskers SEM, *n*=4 biologically independent experiments. *P*-values were calculated by comparing G2/M cell cycle distributions at 72h post-release. g) Time frames (in min) from time-lapse microscopy showing RDES EwS cells with/without *PRC1* knockdown exhibiting mitotic activity without/with subsequent cytokinesis related to Fig. 3h. Arrow indicates ectopic furrow formation in *PRC1* knockdown cells. Cells were stained with Cy5™/Deep Red-F-Actin (red) and counterstained with Hoechst DNA dye (blue). Arrows indicate non-evenly segregated chromosome or merged single nuclei. Scale bar=10 µm. Two-sided Mann-Whitney test in all panels.

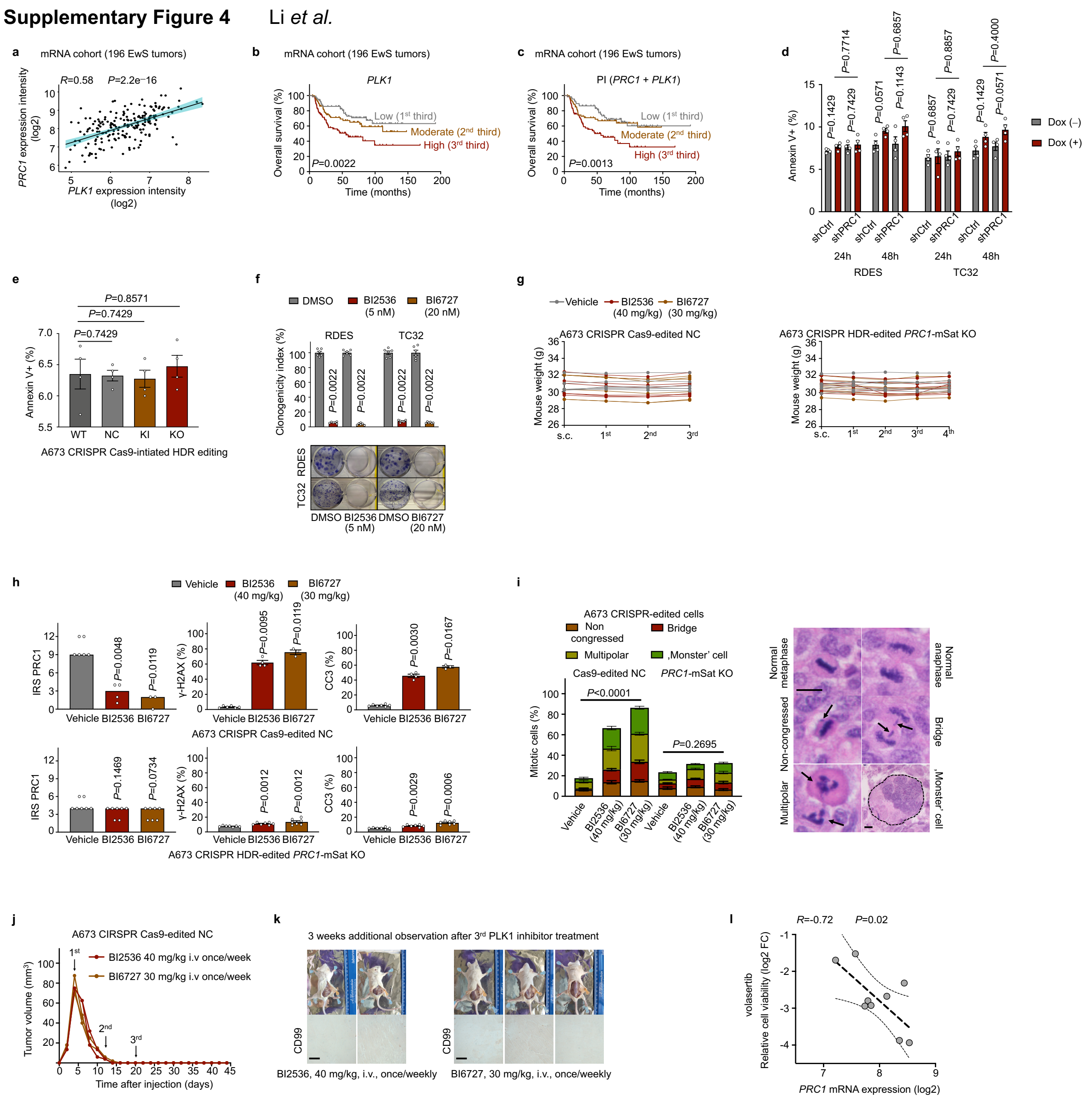

Supplementary Fig. 4 | EwS is sensitive to PLK1 inhibitor treatment

a) Correlation of *PLK1* and *PRC1* expression in EwS tumors. Two-sided test. b) Kaplan-Meier analysis of EwS patients stratified by thirds of *PLK1* expression. Two-sided Mantel-Haenszel test. c) Kaplan-Meier analysis of EwS patients stratified by thirds of combined prognostic index. Two-sided Mantel-Haenszel test. d) Analysis of apoptosis in EwS cells harboring Dox-inducible shPRC1 or shCtrl at 24h and 48h after *PRC1* knockdown. Dots represent means and whiskers SEM, n=4 biologically independent experiments. Two-sided Mann-Whitney test. e) Analysis of apoptosis in wt and CRISPR Cas9-initiated HDR edited A673 cells 24h after seeding. Dots represent means and whiskers SEM, n=4 biologically independent experiments. Two-sided Mann-Whitney test. f) Colony-forming of EwS cells treated with BI2536 or BI6727. Data are displayed as individual dots. Horizontal bars represent means and whiskers SEM, n=6 biologically independent experiments; Two-sided Mann-Whitney test. g) Mice body weight during treatment. Dots represent individual mice, n=6 animals per condition for NC-cells, n=7 animals per condition for KO-cells. h) Quantification of IRS of PRC1 and positivity of γ-H2AX and cleaved caspase-3 (CC3) of xenografts (Fig. 4f). Data are displayed as individual dots. Horizontal bars represent median IRS of PRC1 or means and whiskers

SEM for positivity, respectively; *n* indicates the number of animals per condition: NC-cells, *n*=6 vehicle, *n*=4 BI2536, *n*=3 BI6727; *n*=7 for all conditions in KO-cells; Two-sided Mann-Whitney test. i) Representative micrographs of xenografts stained with H&E (Fig. 4f) and quantification of mitotic defects (min. 30 evaluated mitoses per tumor). Horizontal bars represent means and whiskers SEM, the number of independent animals per condition is as in (h); two-sided Chi-squared test; scale bar=10 µm. j) Volume of xenografts during PLK1 inhibition and 25d of follow-up. k) Upper: Images of necropsy on xenografts showing complete tumor regression. Lower: Immunohistological assessment of the injection site stained with the EwS-marker CD99. Scale bar=100 µm. l) Correlation analysis of *PRC1* mRNA expression level with relative cell viability upon PLK1 inhibitor BI6727 (Volasertib) treatment in 9 *EWSR1-FLI1* positive EwS cell lines derived from DepMap project (data censoring: 25th May 2021). One-sided test.

Supplementary Figure 5 Li *et al.*

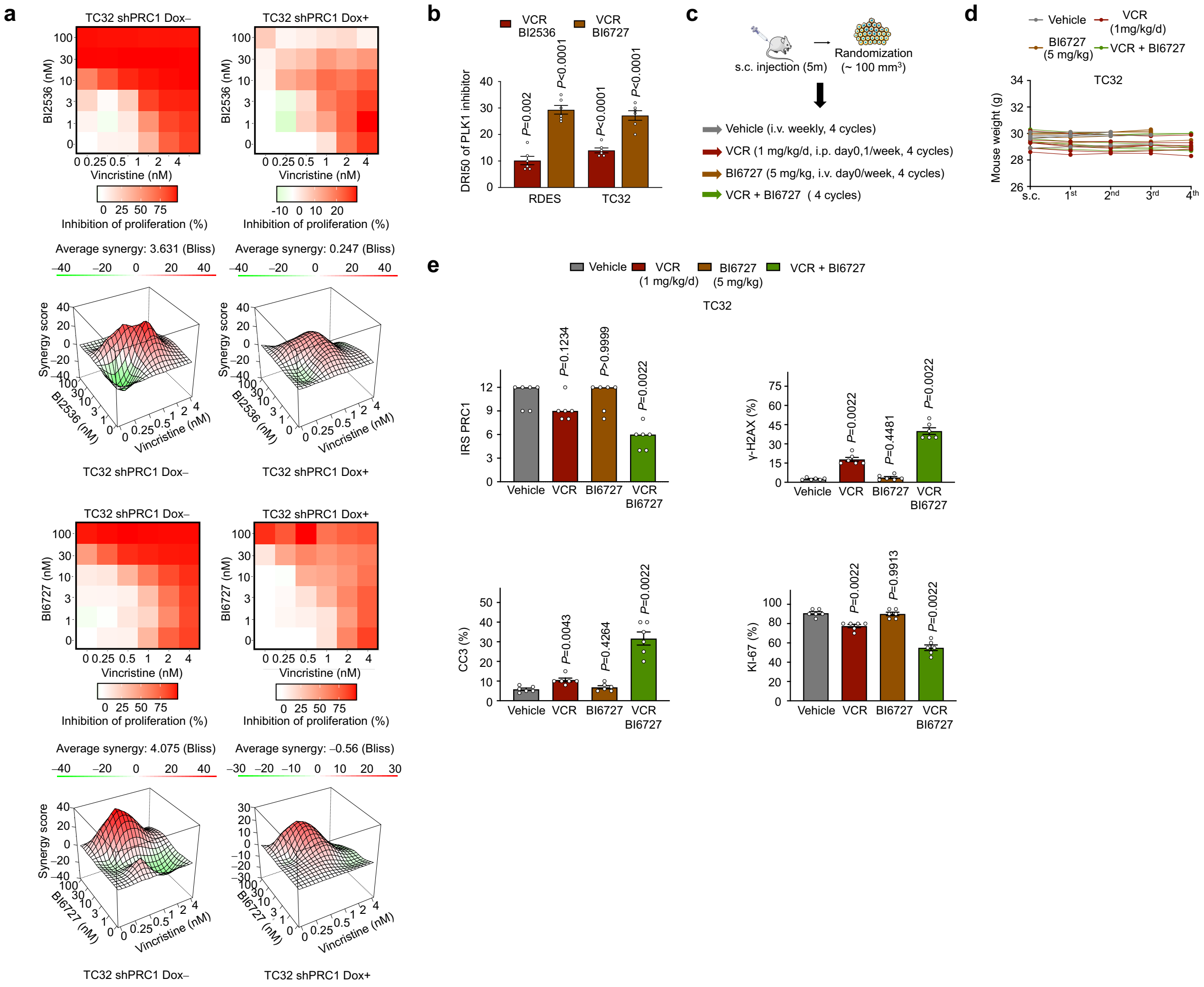

Supplementary Fig. 5 | Combining PLK1 inhibitors with vincristine in EwS cells

a) Excess over Bliss analysis of TC32 EwS cells with/without shRNA-mediated *PRC1* knockdown treated with combinations of vincristine (VCR) and the PLK1 inhibitors BI2536 or BI6727 (red color indicates synergy) *in vitro*. Summary level data (means) from *n*=6 biologically independent experiments are shown. b) Dose reduction index (DRI) of PLK1 inhibitor BI2536 or BI6727 at IC50 level when combined with VCR in RDES and TC32 EwS cells. Data are displayed as individual dots. Horizontal bars represent means and whiskers SEM, *n*=6 biologically independent experiments. c) Scheme of the experimental setting of VCR and PLK1 inhibitor combination treatment (BI6727) *in vivo*. NSG mice were xenografted with TC32 EwS cells. When tumors were palpable, mice were randomized and treated with either vehicle or VCR alone in a dose of 1 mg/kg/d or PLK1 inhibitor BI6727 in a dose of 5 mg/kg or in combination. d) Body weight of mice during intraperitoneal and/or intravenous VCR and/or PLK1 inhibitor BI6727 treatments. Dots represent means and whiskers SEM, *n*=6 animals per condition. e) Quantification of IRS of PRC1 and positivity of γ-H2AX, cleaved caspase-3 (CC3) and Ki-67 per HPF of xenografts related to Fig. 4h. Data are displayed as individual dots. Horizontal bars represent median IRS of PRC1 or means and whiskers SEM for positivity, respectively; *n*=6 animals per condition; two-sided Mann-Whitney test.

Supplementary Figure 6 Li *et al.*

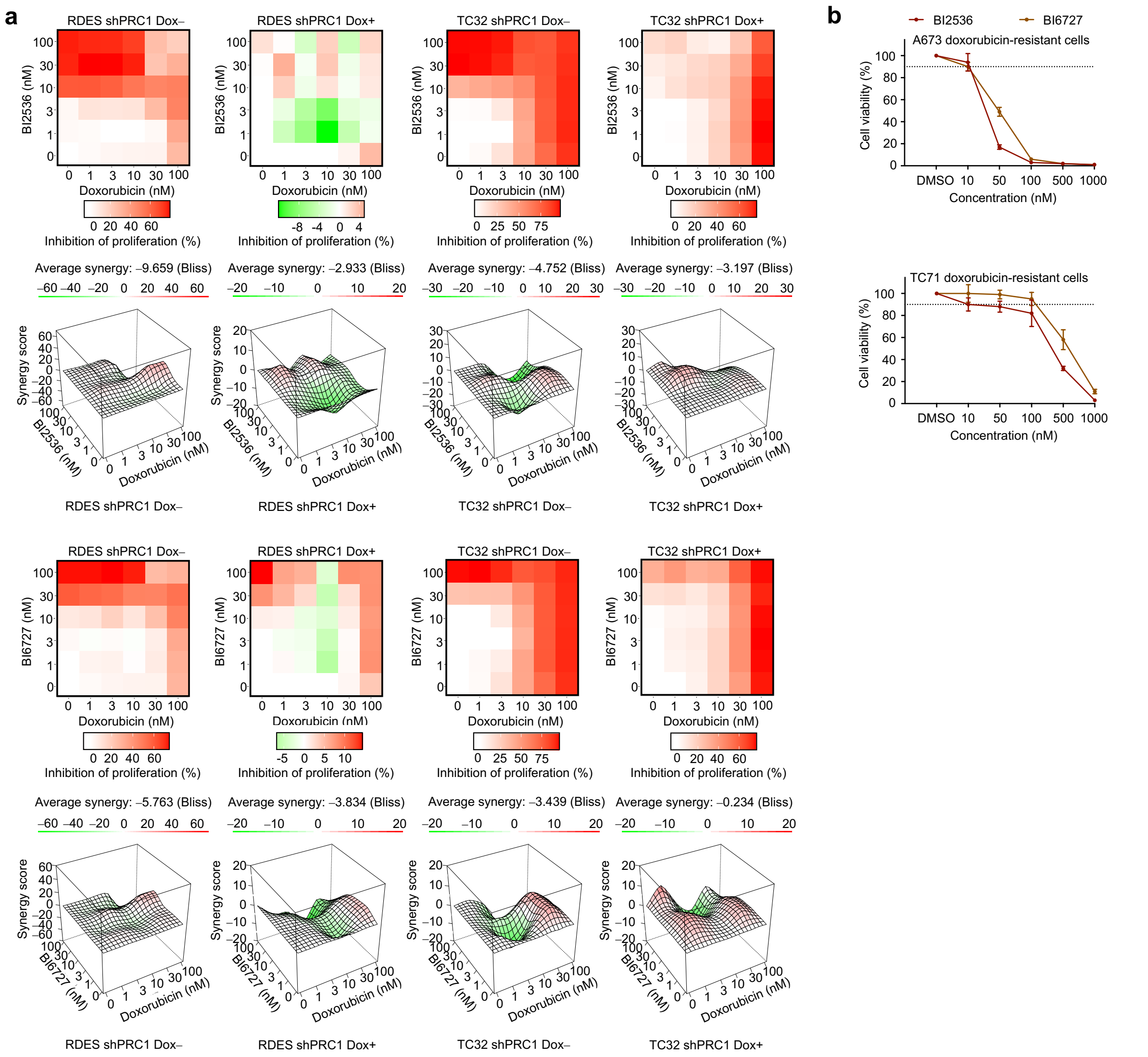

Supplementary Fig. 6 | PLK1 inhibitor treatment in doxorubicin-resistant EwS cells
a) Excess over Bliss analysis of RDES and TC32 EwS cells with/without *PRC1* knockdown treated with combinations of doxorubicin and the PLK1 inhibitors BI2536 or BI6727 (red color indicates synergy) *in vitro*. Summary level data (means) from *n*=6 biologically independent experiments are shown. b) Dose response of two doxorubicin-resistant (Doxo-res) EwS cell lines (A673, TC71) treated with the PLK1 inhibitors BI2536 or BI6727. Concentration of either PLK1 inhibitor below inhibitory concentrations of 90% viability (IC10) was defined as non-toxic (dashed lines). Dots represent means and whiskers SEM, *n*=6 biologically independent experiments.

Supplementary Figure 7 Li *et al.*

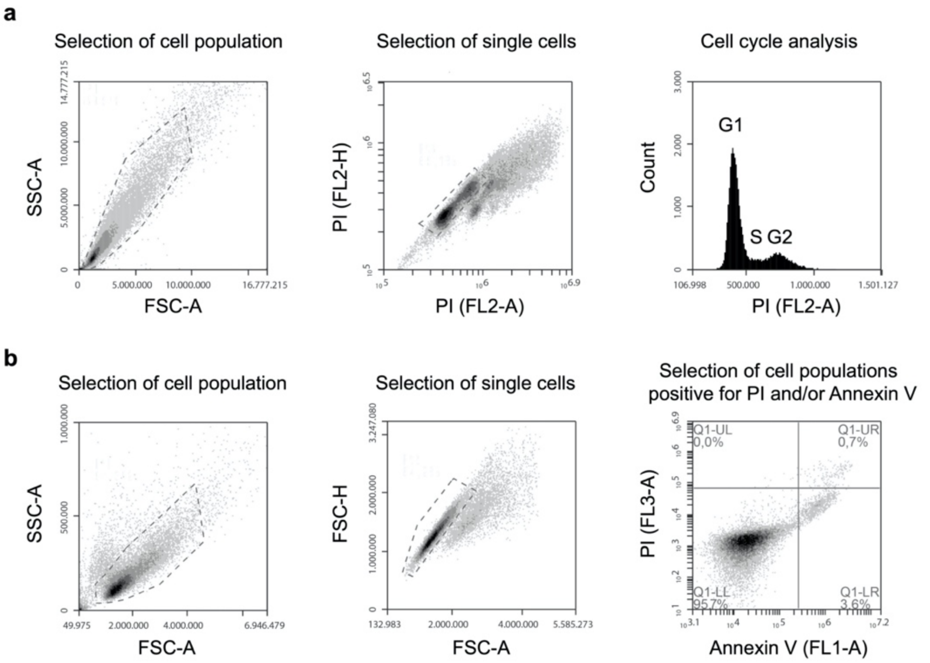

Supplementary Fig. 7 | Examples of flow-cytometric gating strategies
a) Gating strategy for flow-cytometric cell-cycle analysis of EwS cells using PI. b) Gating strategy for Annexin V/PI staining of EwS cells.
