# Supplementary material for: Therapeutic targeting of the PLK1-PRC1-axis triggers cell death in genomically silent childhood cancer
Source: Nat Commun. 2021 Sep 16;12:5356. doi: 10.1038/s41467-021-25553-z (PMC8445938; doi:10.1038/s41467-021-25553-z)
Supplement: Supplementary file 3 — Description of Additional Supplementary Files [file 41467_2021_25553_MOESM3_ESM.docx]

Description of Additional Supplementary Files

Title: Supplementary Data file 1

Description: Evaluation of risk-factors of prognosis in 96 primary EwS patients by multivariate Cox regression analysis

Title: Supplementary Data file 2

Description: Transcriptome profiling data from EwS cell lines with/without Dox-induced silencing of PRC1 compared to a negative control shRNA

Title: Supplementary Data file 3

Description: Summary of clinical trials for BI2536 and BI6727 (data censoring 20th October 2020)

Title: Supplementary Data file 4

Description: Interpretation of PLK1 inhibitor mouse dose to human equivalent dose transversion

Title: Supplementary Data file 5

Description: Analysis of matched in vivo gene expression and drug-response data from pediatric tumor types with relatively silent genomes

Title: Supplementary Data file 6

Description: Analysis of mRNA expression of PRC1, PLK1, and MKI67 and PLK1 inhibitor BI6727 (Volasertib) drug-response data in EwS cell lines from the DepMap project

Title: Supplementary Data file 7

Description: Correlation analysis of STAG2 and/or TP53 mutation status with PRC1 expression level (RNA seq) in 57 primary EwS tumors

Title: Supplementary Data file 8

Description: List of oligonucleotide sequences

Title: Supplementary Data file 9

Description: GSEA results for weighted correlation network analysis upon PRC1 knockdown
