# Supplementary material for: Therapeutic targeting of the PLK1-PRC1-axis triggers cell death in genomically silent childhood cancer
Source: Nat Commun. 2021 Sep 16;12:5356. doi: 10.1038/s41467-021-25553-z (PMC8445938; doi:10.1038/s41467-021-25553-z)
Supplement: Supplementary file 13 — Reporting Summary [file 41467_2021_25553_MOESM13_ESM.pdf]

nature research

Corresponding author(s): Thomas G. P. Grünewald

Last updated by author(s): Aug 6, 2021

Reporting Summary

Nature Research wishes to improve the reproducibility of the work that we publish. This form provides structure for consistency and transparency in reporting. For further information on Nature Research policies, see our Editorial Policies and the Editorial Policy Checklist.

Statistics

For all statistical analyses, confirm that the following items are present in the figure legend, table legend, main text, or Methods section.

n/a	Confirmed	
☐	☒	The exact sample size (*n*) for each experimental group/condition, given as a discrete number and unit of measurement
☐	☒	A statement on whether measurements were taken from distinct samples or whether the same sample was measured repeatedly
☐	☒	The statistical test(s) used AND whether they are one- or two-sided<br>*Only common tests should be described solely by name; describe more complex techniques in the Methods section.*
☐	☒	A description of all covariates tested
☐	☒	A description of any assumptions or corrections, such as tests of normality and adjustment for multiple comparisons
☐	☒	A full description of the statistical parameters including central tendency (e.g. means) or other basic estimates (e.g. regression coefficient) AND variation (e.g. standard deviation) or associated estimates of uncertainty (e.g. confidence intervals)
☐	☒	For null hypothesis testing, the test statistic (e.g. *F*, *t*, *r*) with confidence intervals, effect sizes, degrees of freedom and *P* value noted<br>*Give P values as exact values whenever suitable.*
☒	☐	For Bayesian analysis, information on the choice of priors and Markov chain Monte Carlo settings
☒	☐	For hierarchical and complex designs, identification of the appropriate level for tests and full reporting of outcomes
☐	☒	Estimates of effect sizes (e.g. Cohen's *d*, Pearson's *r*), indicating how they were calculated

Our web collection on statistics for biologists contains articles on many of the points above.

Software and code

Policy information about availability of computer code

Data collection	GraphPad PRISM 8, Microsoft Excel (v16.16.5), CFX Manager (v3.1), BD Accuri C6 Cytometer (v.227), ZEN pro (v2.3), AxioVision (v4.9)
Data analysis	GraphPad PRISM 8, Microsoft Excel (v16.16.5), CFX Manager (v3.1), BD Accuri C6 Cytometer (v.227), Transcriptome Analysis Console v4.0, Clariom D Array version 2, R project (4.0.2), SPSS (v.23.0), Adobe Photoshop 2020, Image J (v1.8.0_172), CompuSyn (v1.0.1)

For manuscripts utilizing custom algorithms or software that are central to the research but not yet described in published literature, software must be made available to editors and reviewers. We strongly encourage code deposition in a community repository (e.g. GitHub). See the Nature Research guidelines for submitting code & software for further information.

Data

Policy information about availability of data

All manuscripts must include a data availability statement. This statement should provide the following information, where applicable:

- Accession codes, unique identifiers, or web links for publicly available datasets
- A list of figures that have associated raw data
- A description of any restrictions on data availability

The microarray data were deposited at the National Center for Biotechnology Information (NCBI) GEO database under the accession code GSE156559. The microarray, DNase-Seq, and ChIP-Seq data referenced during the study are available in a public repository from the GEO website (https://www.ncbi.nlm.nih.gov/geo/); accession codes: GSM736570 and GSE61944. The source data underlying Figs. 1a–c, 2a–g, 3a–j, 4a–j and Supplementary Figs. 1, 2b–h, 3a–g, 4a–h, 5a–e, 6a-b are provided as a Source Data file. All the other data supporting the findings of this study and custom code are available within the article and its supplementary information files and/or from the corresponding author upon reasonable request.

April 2020

Field-specific reporting

Please select the one below that is the best fit for your research. If you are not sure, read the appropriate sections before making your selection.

☒ Life sciences ☐ Behavioural & social sciences ☐ Ecological, evolutionary & environmental sciences

For a reference copy of the document with all sections, see nature.com/documents/nr-reporting-summary-flat.pdf

Life sciences study design

All studies must disclose on these points even when the disclosure is negative.

Sample size	In vitro sample sizes were chosen empirically based on previous similar studies that have given statistical results (Marchetto A et al. 2020 Nature Communications, PMID: 32415069; Musa J et al. 2019 Nature Communications, PMID: 31511524). For in vivo experiments, sample sizes were predetermined using power calculations with beta=0.8 and alpha<0.05 based on preliminary data and in compliance with the 3R system (replacement, reduction, refinement).
Data exclusions	No data were excluded from the analyses.
Replication	All attempts at replication yielded similar results. Precise information on the number of biologically independent replicates of each experiment are given in the corresponding figure legends.
Randomization	For in vivo experiments, mice were numbered and randomized into control and treatment groups with similar distributions of male and female animals. All mice had a similar age at start of the given experiment (10-12 weeks). For in vitro experiments, randomization and controlling of covariates was not necessary or advised, since the highly standardized cell culture conditions (see Methods section) largely eliminate biological noise/variation across experiments.
Blinding	Histopathological analysis of human tissue samples was carried out in a blinded fashion. For animal treatment studies, drug administration was only possible in an unblinded manner due to ethical regulations and monitoring of potential adverse effects. For histopathological assessment of the xenografts, the investigators were blinded with respect to group allocation during data collection and analysis. For all other experiments than those listed here, the investigators were not blinded to group allocation, because it was either not relevant or since this could have caused errors in applying the correct treatment/experimental conditions after group assignment. However investigators have been blinded during data collection and/or analysis.

Reporting for specific materials, systems and methods

We require information from authors about some types of materials, experimental systems and methods used in many studies. Here, indicate whether each material, system or method listed is relevant to your study. If you are not sure if a list item applies to your research, read the appropriate section before selecting a response.

Materials & experimental systems

n/a	Involved in the study	
☐	☒	Antibodies
☐	☒	Eukaryotic cell lines
☒	☐	Palaeontology and archaeology
☐	☒	Animals and other organisms
☐	☒	Human research participants
☒	☐	Clinical data
☒	☐	Dual use research of concern

Methods

n/a	Involved in the study	
☒	☐	ChIP-seq
☐	☒	Flow cytometry
☒	☐	MRI-based neuroimaging

Antibodies

Antibodies used	For immunohistochemistry analyses, the following antibodies were used:<br>Polyclonal rabbit anti-PRC1 (Proteintech, clone ID not applicable, cat-no 15617-1-AP, dilution:1:200), polyclonal rabbit-anti-cleaved caspase 3 (Cell Signaling, clone ID not applicable, cat-no 9661, dilution:1:100), monoclonal rabbit-anti-Ki-67 (Cell Marque, clone ID SP-6, cat-no 275R-15, dilution:1:200), monoclonal rabbit-anti-phospho-gammaH2AX (Abcam, clone ID EP854(2)Y, cat-no ab81299, dilution:1:8,000), monoclonal mouse-anti-human mitochondria (Abcam, clone ID 113-1, cat-no ab92824, dilution:1:1,000), monoclonal mouse-anti-CD99 (Abcam, clone ID 12E7, cat-no ab8855, dilution:1:40), polyclonal anti-mouse IgG (ImmPress, clone ID not applicable, cat-no MP-7402, dilution: not applicable since commercial ready-to-use solution), polyclonal anti-rabbit IgG (ImmPress, clone ID not applicable, cat-no MP-7401, dilution: not applicable since commercial ready-to-use solution), Dako REAL™ Detection System, Alkaline Phosphatase/RED,Rabbit/Mouse (Dako, clone ID not applicable, cat-no K5005, dilution: not applicable since commercial ready-to-use solution).<br><br>For western blot analyses, the following antibodies were used:

Polyclonal rabbit-anti-PRC1 (Proteintech, clone ID not applicable, cat-no 15617-1-AP, dilution:1:1,000), monoclonal mouse-anti-GAPDH (Santa Cruz, clone ID 6C5, cat-no sc-32233, dilution:1:800), polyclonal anti-rabbit IgG (H+L) (OriGene, clone ID not applicable, cat-no R1364HRP, dilution:1:5,000), polyclonal anti-mouse IgG (H+L) (Promega, clone ID not applicable, cat-no W402B, dilution:1:3,000).

Validation

Specificity of the used antibodies against human antigens was tested by knockdown experiments of the corresponding antigen and subsequent Western blot and/or immunohistochemistry analyses. In addition, antibodies were validated for the given application by the manufacturer as stated in the respective datasheets (see links) and references (see DOIs):

* Polyclonal rabbit anti-PRC1 (Proteintech, clone ID not applicable, cat-no 15617-1-AP): https://www.ptglab.com/products/PRC1-Antibody-15617-1-AP.htm, Ref: DOI: 10.1111/gtc.12803

* Polyclonal rabbit-anti-cleaved caspase 3 (Cell Signaling, clone ID not applicable, cat-no 9661), https://www.cellsignal.de/products/primary-antibodies/cleaved-caspase-3-asp175-antibody/9661, Ref: DOI:10.3892/ijmm.2020.4453

* Monoclonal rabbit-anti-Ki-67 (Cell Marque, clone ID SP-6, cat-no 275R-15), https://www.cellmarque.com/antibodies/CM/108/Ki-67_SP6, Ref: DOI:10.1097/00005072-199810000-00005

* Monoclonal mouse-anti-GAPDH (Santa Cruz, clone ID 6C5, cat-no sc-32233), https://datasheets.scbt.com/sc-32233.pdf, Ref: DOI:10.1016/j.yjmcc.2019.01.029

* Polyclonal anti-rabbit IgG (H+L) (OriGene, clone ID not applicable, cat-no R1364HRP), https://cdn.origene.com/datasheet/r1364hrp.pdf, Ref: DOI:10.2164/jandrol.109.008623

* Polyclonal anti-mouse IgG (H+L) (Promega, clone ID not applicable, cat-no W402B), https://www.promega.de/products/protein-detection/primary-and-secondaryantibodies/anti_mouse-igg-h-and-l-hrp-conjugate/?catNum=W4021#specifications , Ref: DOI:10.1074/jbc.M112.343566

* Monoclonal rabbit-anti-phospho-gammaH2AX (Abcam, clone ID EP854(2)Y, cat-no ab81299): https://www.abcam.com/gamma-h2ax-phospho-s139-antibody-ep8542y-ab81299.html, Ref: DOI: 10.1155/2015/136810

* Monoclonal mouse-anti-human mitochondria (Abcam, clone ID 113-1, cat-no ab92824): https://www.abcam.com/mitochondria-antibody-113-1-ab92824.html. Ref: DOI: 10.1007/s13577-017-0198-2

* Monoclonal mouse-anti-CD99 (Abcam, clone ID 12E7, cat-no ab8855): https://www.agilent.com/en/product/immunohistochemistry/antibodies-controls/primary-antibodies/cd99-mic2-gene-products-ewing-s-sarcoma-marker-(autostainer-autostainer-plus) 76403#productdetails, Ref: DOI: 10.3390/cancers12030644

* Polyclonal anti-mouse IgG (ImmPress, clone ID not applicable, cat-no MP-7402): https://vectorlabs.com/immpress-hrp-horse-anti-mouse-igg-peroxidase-polymer-detection-kit.html, Ref: DOI: 10.1038/s41467-020-17985-w

* Polyclonal anti-rabbit IgG (ImmPress, clone ID not applicable, cat-no MP-7401): https://vectorlabs.com/immpress-hrp-horse-anti-rabbit-igg-peroxidase-polymer-detection-kit.html, Ref: DOI: 10.1186/s40170-020-00212-x

* Dako REAL™ Detection System, Alkaline Phosphatase/RED,Rabbit/Mouse (Dako, clone ID not applicable, cat-no K5005, dilution: ready-to-use): https://www.agilent.com/store/en_US/Prod-K500511-2/K500511 2?navAction=push&catId=SubCat3ECS_86714&pCatName=Other%20Visualization%20Systems&navCount=0, Ref: DOI: 10.1038/s41598-017-13888-x

Eukaryotic cell lines

Policy information about cell lines

Cell line source(s)

The A673, HEK293T, and RDES were purchased from ATCC. Human Ewing sarcoma cell lines were provided by the following repositories and/or sources: A673 cells were purchased from ATCC. SK-N-MC was provided by the German Collection of Microorganism and Cell Cultures (DSMZ). TC32 and TC71 were kindly provided by the Children's Oncology Group (COG), and EW1 was provided by O. Delattre (Paris, France). A673/TR/shEF1 cells were provided by J. Alonso (Madrid, Spain).

Authentication

Cell lines were authenticated by STR-profiling and by detection of specific fusion oncogenes by qRT-PCR.

Mycoplasma contamination

All cell lines were tested negative for mycoplasma contamination by nested PCR.

Commonly misidentified lines
(See ICLAC register)

SK-N-MC: The SK-N-MC cell line is listed in the database of commonly misidentified cell lines, ICLAC (http://iclac.org/databases/cross-contaminations), as it was initially described to be a neuroblastoma cell line. Indeed, it is a Ewing sarcoma cell line expressing the pathognomonic fusion oncogene EWSR1-FLI1. This definitive and established Ewing sarcoma cell line was used in this study because it displayed appropriate expression levels of the gene of interest of this study (PRC1).

Animals and other organisms

Policy information about studies involving animals; ARRIVE guidelines recommended for reporting animal research

Laboratory animals	NOD.Cg-Prkdcscid Il2rgtm1Wjl/SzJ (NSG) mice were obtained from Jackson Laboratory via Charles River Laboratories and bred under an MTA with LMU Munich. All NSG mice (male and female) had an age between 10 and 12 weeks at start of the experiments. Mice were housed in individually ventilated cages (IVC) under specific pathogen-free (SPF) conditions with strict dark/light cycles (darkness from 6 PM to 6 AM), an ambient temperature of 21-23°C and a humidity of 45-65%.
Wild animals	No wild animals were used in this study.
Field-collected samples	No field-collected samples were used in the study.
Ethics oversight	Experiments were approved by the governments of Upper Bavaria and Northbaden, and conducted in accordance with ARRIVE guidelines, recommendations of the European Community (86/609/EEC), and UKCCCR (guidelines for the welfare and use of animals in cancer research).

Note that full information on the approval of the study protocol must also be provided in the manuscript.

Human research participants

Policy information about studies involving human research participants

Population characteristics	For survival analyses, public microarray data of 196 primary Ewing sarcoma tumors (accession codes: GSE63157, GSE34620, GSE12102, GSE17618, and unpublished) for which clinical annotations were available were downloaded and processed as indicated in the cited reference. For the TMA-cohort, Ewing sarcoma tumors were collected with informed consent from patients enrolled in national Ewing sarcoma trials coordinated by the EWING trial center at the University of Essen (Germany). Overall, the patients' clinical features and demographics were typical of Ewing sarcoma. The mean age at diagnosis was 20.1 years, the ratio of males/females was 1.4.
Recruitment	Human FFPE tissue samples were retrieved from the archives of the Institute of Pathology of the LMU Munich (Germany) or the Gerhard-Domagk Institute of Pathology of the University of Münster (Germany) comprising the years 2008-2018. All available samples for the given tumor entity were collected and screened by a pathologist for sufficient material on histological slides. Since the observed expression pattern of PRC1 at the protein level in these samples (Fig. 1c) was highly similar to the PRC1 mRNA expression pattern observed in well-curated and publicly available gene expression microarray data (Fig. 1a, Supplementary Fig. 1) (Baldauf MC et al. 2008 OncoImmunology), selection bias appears unlikely.
Ethics oversight	Human FFPE tissue samples were retrieved from the archives of the Institute of Pathology of the LMU Munich (Germany) or the Gerhard-Domagk Institute of Pathology of the University of Münster (Germany) with approval of the institutional review boards. All patients gave informed consent. Tissue-microarrays (TMAs) were stained and analyzed with approval of the ethics committee of the LMU Munich (approval no. 550-16 UE).

Note that full information on the approval of the study protocol must also be provided in the manuscript.

Flow Cytometry

Plots

Confirm that:

- [x] The axis labels state the marker and fluorochrome used (e.g. CD4-FITC).
- [x] The axis scales are clearly visible. Include numbers along axes only for bottom left plot of group (a 'group' is an analysis of identical markers).
- [x] All plots are contour plots with outliers or pseudocolor plots.
- [x] A numerical value for number of cells or percentage (with statistics) is provided.

Methodology

Sample preparation	Human Ewing sarcoma cells were trypsinized from cell culture dishes, washed with PBS, and stained for AnnexinV-FITC and propidium iodine (PI) using the BD Pharmingen Apoptosis detection kit I according to the manufacturer's protocol.
Instrument	Accuri C6 flow cytometer
Software	Accuri C6 CFlow plus
Cell population abundance	At least 1*10e5 events were counted per assay.
Gating strategy	Gating included forward and sideward scatter plots for gating living cells (i.e. exclusion of debris), and subsequent filtering for single cells.

- [x] Tick this box to confirm that a figure exemplifying the gating strategy is provided in the Supplementary Information.
